# A randomized controlled trial comparing the clinical efficacy and cost-effectiveness of eye movement desensitization and reprocessing (EMDR) and integrated EMDR-Dialectical Behavioural Therapy (DBT) in the treatment of patients with post-traumatic stress disorder and comorbid (Sub)clinical borderline personality disorder: study design

**DOI:** 10.1186/s12888-020-02713-x

**Published:** 2020-08-06

**Authors:** Aishah Snoek, Aartjan T. F. Beekman, Jack Dekker, Inga Aarts, Gerard van Grootheest, Matthijs Blankers, Chris Vriend, Odile van den Heuvel, Kathleen Thomaes

**Affiliations:** 1 Arkin Sinai Centrum, Amstelveen, The Netherlands; 2 Amsterdam UMC, University of Amsterdam, Department of Psychiatry, Amsterdam, the Netherlands; 3 Amsterdam UMC, Vrije Universiteit Amsterdam, Psychiatry, Amsterdam Neuroscience, Amsterdam, the Netherlands; 4 Amsterdam UMC, Vrije Universiteit Amsterdam, Anatomy and Neurosciences, Amsterdam Neuroscience, Amsterdam, the Netherlands; 5 GGZinGeest, Department of Psychiatry, Amsterdam, the Netherlands; 6 Arkin Mental Health Care, Amsterdam, the Netherlands; 7 Vrije Universiteit Amsterdam, Faculty of Behavioural and Movement Sciences, Amsterdam, the Netherlands; 8 Trimbos Institute, Institute of Mental Health and Addiction, Utrecht, the Netherlands

**Keywords:** EMDR, PTSD, Dialectical behaviour therapy, Borderline personality disorder

## Abstract

**Background:**

Comorbidity between Posttraumatic Stress Disorder (PTSD) and Borderline Personality Disorder (BPD) is high. There is growing motivation among clinicians to offer PTSD treatments – such as Eye Movement Desensitization and Reprocessing (EMDR) - to patients with PTSD and comorbid BPD. However, a large subgroup with comorbid BPD does not sufficiently respond to PTSD treatment and is more likely to be excluded or to dropout from treatment. Dialectical Behaviour Therapy (DBT) for BPD is well established and although there is some evidence that DBT combined with DBT Prolonged Exposure (DBT + DBT PE) is twice as effective in reducing PTSD symptoms than DBT alone, the comparative efficacy of integrated PTSD-DBT and PTSD-only treatment has not been investigated yet. The current study will therefore evaluate the comparative clinical efficacy and cost-effectiveness of EMDR-DBT and EMDR-only in patients with PTSD and comorbid (sub)clinical BPD. Moreover, it is not clear yet what treatment works best for which individual patient. The current study will therefore evaluate neurobiological predictors and mediators of the individual response to treatment.

**Method:**

A randomized controlled trial comparing the clinical efficacy and cost-effectiveness of integrated EMDR-DBT (*n* = 63) and EMDR-only (n = 63) in treatment-seeking adult patients with PTSD and comorbid (sub)clinical BPD. In addition, neurobiological predictors and mediators of treatment outcome, such as hair cortisol, FKBP5 and BDNF protein levels and FKBP5 and BDNF methylation status, are measured through hair and blood samples.

**Discussion:**

This is the first study to compare the clinical efficacy and cost-effectiveness of integrated EMDR-DBT and EMDR-only in patients with PTSD and comorbid (sub)clinical BPD, while simultaneously identifying individual predictors and mediators of treatment response. Results will reveal which treatment works best for which individual patient, thereby guiding individual treatment choices and personalizing psychiatry.

****Trial registration**:**

Clinical Trials, NCT03833453. Retrospectively registered, 15 March 2019.

## Background

Psychologically traumatic events like terrorist acts, civil wars, community violence, and natural disasters are highly prevalent in people’s lives. During life, 90% of the population experiences at least one traumatic event [1]. The posttraumatic stress often resulting from such events poses a significant public health challenge. With a lifetime prevalence of 10%, posttraumatic stress disorder (PTSD) is one of the most prevalent DSM-5 disorders [1]. Symptoms include intrusive memories and nightmares of the trauma, irritability, hyper vigilance, difficulty sleeping, poor concentration and emotional withdrawal (DSM-5, 2013). In addition to the clinical burden, many patients experience relational, occupational and financial problems [2]. On top of that, PTSD is associated with greater mental health utilization and higher health care costs, including costs for specialist and primary care [3, 4]. Consequences of PTSD are thus costly, not only for patients but also to the health care system and to society as a whole. The high prevalence along with the significant functional and financial burden makes research into clinically- and cost-effective treatments for PTSD imperative [5].

Eye movement desensitization and reprocessing (EMDR) is a widely used intervention for the treatment of PTSD [6]. Although the mechanisms underlying EMDR have been a source of controversy, its efficacy for PTSD is now generally supported [7]. More than half of patients who complete EMDR treatment significantly improve compared to usual-care or waiting list [8, 9]. However, despite the well-established efficacy of EMDR for PTSD, a large subgroup of patients does not sufficiently respond to treatment [10, 11]. There is some evidence that patients with a PTSD and comorbid BPD symptoms are less likely to achieve good end-state functioning after trauma-focused treatment than those without comorbid BPD symptomatology ([12–14] – in preparation). Moreover, comorbidity between PTSD and BPD might be associated with higher dropout from PTSD treatment than PTSD alone [15]. Lastly, because of the common confluence of exclusion criteria for suicidality and self-destructive behaviour, a large subgroup of patients with comorbid borderline personality features is excluded from PTSD treatment [16].

Comorbidity between PTSD and borderline personality disorder (BPD) is high, with at least a quarter of PTSD patients also meeting BPD criteria and vice versa [17]. BPD is characterised by a pervasive pattern of instability in self-image, affect regulation and interpersonal relationships [18]. The disorder causes severe psychological impairment and is associated with a high mortality rate due to suicide [19]. This is also true for patients with subclinical BPD, who are diagnosed with a higher number of DSM 5 disorders, have higher marginal levels of suicide attempts and experience more social and professional problems compared to healthy controls [20]. Dialectical Behaviour Therapy (DBT) is a frequently investigated psychological intervention for BPD [21, 22]. DBT significantly reduces self-mutilation and suicidal behaviour, while improving treatment compliance in both subclinical and clinical BPD [23]. In line with this, DBT as a precursor to PTSD treatment reduces suicidal and self-injuring behaviour in PTSD patients with comorbid BPD, thereby increasing the inclusion rate for subsequent PTSD treatment [24]. However, given the severe physical and emotional distress, it is far from ideal to have a patient wait for 12 months before starting PTSD treatment. Besides, successive DBT and EMDR treatment could take up to 18 months, which does not constitute the most time efficient alternative and may lead to patients dropping out from treatment prematurely. Simultaneously treating PTSD and BPD might thus provide a more time-efficient alternative, given that such an integrated treatment would result in considerably shorter treatment duration. Moreover, treating PTSD while simultaneously treating borderline symptomology might reduce exclusion and dropout rates while improving end-state functioning, thereby holding promise for improving clinical efficacy and cost effectiveness at the same time [25–27]. Although two randomized controlled trials have already established the clinical efficacy of integrating DBT with DBT PE as compared to DBT-only [28] and of integrating DBT with trauma-focused cognitive-behavioural approaches as compared to waiting-list [25], much remains to be learned about the clinical efficacy of integrated PTSD-DBT compared to PTSD-only treatment (see also the online study protocol of Bohus et al. [29] who compared an integrated DBT-PTSD treatment, with interventions derived from cognitive behavioural therapy, acceptance and commitment therapy and compassion-focused therapy, to cognitive processing therapy for PTSD). Moreover, although previous studies found DBT to have the highest cost-effectiveness compared to treatment as usual (i.e. weekly individual therapy, supportive or psycho-educational groups) and compared to cognitive behavioural therapy (CBT), the cost-effectiveness of EMDR for PTSD has not been investigated yet, let alone compared to integrated EMDR-DBT [26, 30]. The current study aims to bridge these knowledge gaps by comparing the clinical efficacy and cost-effectiveness of integrated EMDR-DBT to EMDR-only in adults with PTSD and comorbid (sub)clinical BPD. It is hypothesized that clinical efficacy and cost-effectiveness will be higher for integrated EMDR-DBT than for EMDR-only.

Although comparing the clinical efficacy and cost-effectiveness of integrated EMDR-DBT and EMDR-only might lead to novel insights for the treatment of PTSD with comorbid (sub)clinical BPD, solely studying the link between treatments and symptoms would fail to explain how exactly these treatments potentially affects these symptoms. Investigating potential predictors and mediating pathways might aid in the identification of causal mechanisms underlying a certain treatment effect and thereby assist in the modification of elements that are crucial for therapeutic change, while dismissing those found to be redundant. Such variables could thus specify for whom and under which conditions a treatment might or might not be effective, thereby maximizing treatment efficacy and efficiency while minimizing costs.

To the best of our knowledge, to date no randomized controlled trials have been conducted that incorporate potential predictors and mediators of the response to EMDR or DBT treatment in patients with PTSD and BPD respectively. The current study will therefore incorporate three well-known and well-studied neurobiological factors that have been associated with risk, resilience and response to psychotherapy in both PTSD and BPD, thereby linking our primary psychological outcomes to disorder-relevant neurobiological outcomes. As such, the first candidate predictor and mediator constitutes the main stress hormone cortisol. The majority of research supports an enhanced negative feedback inhibition of cortisol on the pituitary gland in individuals with PTSD, resulting in attenuated baseline cortisol levels compared to healthy individuals (for a review see: [31]; Zoladz & Diamond, [26, 32]; for a meta-analysis see: [33]; for a review and meta-analysis of contradicting findings see: [34]). Particularly interesting is that patients with lower initial cortisol levels were found to be more likely to respond to treatment than patients with higher initial levels of cortisol [35]. In addition, patients who responded well to treatment showed an increase in cortisol levels over time, while cortisol levels decreased in non-responders [36]. With regard to BPD findings are less consistent, possibly because of confounding by relevant patient characteristics such as comorbidity with other mental disorders [37]. This hypothesis is supported by previous research demonstrating that cortisol release in BPD depends on the severity of PTSD symptoms, such that more severe PTSD symptoms are associated with reduced cortisol levels [38]. On the other hand, cortisol release in PTSD depends on the presence of BPD, given that veterans with PTSD and comorbid BPD had lower cortisol levels than veterans with PTSD alone [39]. Given that hair cortisol provides a stable, long-term retrospective month-by-month measure of cortisol exposure, the current study will assess whether hair cortisol predicts and mediates the treatment response in patients with PTSD and comorbid BPD [40, 41]. A second candidate predictor and mediator of the treatment response is FKBP5, which is known for regulating cortisol-binding affinity and has been associated with both PTSD and BPD [42, 43]. Reduced FKBP5 protein levels, which are associated with enhanced FKBP5 methylation, facilitate enhanced glucocorticoid receptor (GR) responsiveness, leading to lower cortisol levels in individuals with PTSD and BPD [35, 44–46]. Of particular interest is a study of Yehuda et al. [47], demonstrating that within a sample of PTSD patients, those who responded to treatment showed a decrease in FKBP5 methylation, whereas non-responders showed an increase in FKBP5 methylation. The same result was found in PTSD patients receiving a meditation intervention, indicating that FKBP5 protein levels and FKBP5 methylation status are viable biological correlates of PTSD and BPD and might therefore hold promise as predictors and mediators of the response to treatment [48]. A third candidate predictor and mediator of the treatment response is Brain Derived Neurotrophic Factor (BDNF). A recent study suggested that attenuated BDNF protein levels, which are associated with enhanced BDNF methylation, might contribute to the onset and maintenance of both PTSD and BPD within the same individual [49]. In line with this, Perroud et al. [50] found enhanced BDNF methylation baseline levels in BPD compared to controls, and even higher BDNF methylation baseline levels in patients who experienced a higher number of childhood trauma [50]. Importantly, BPD patients who positively responded to DBT showed a decrease in BDNF methylation over time, whereas non-responders showed an increase in BDNF methylation status [50]. Last of all, Park et al. [51] found that BDNF protein baseline levels might also contribute to the therapeutic response of PTSD patients receiving EMDR, given that BDNF protein baseline levels of responders were higher compared to non-responders [51].

In conclusion, previous research demonstrated that cortisol, FKBP5 and BDNF protein levels and FKBP5 and BDNF methylation status are viable biological correlates of the (change in) PTSD and BPD symptoms. The current study will examine whether these factors can also predict and mediate the response to treatment in patients with PTSD and comorbid (sub)clinical BPD. Given that previous research investigated these biomarkers at the group-level, the current study will assess whether these biomarkers can also predict and mediate the individual treatment response. It is hypothesized that cortisol, FKBP5 and BDNF protein levels and FKBP5 and BDNF methylation status predict and mediate the individual response to integrated EMDR-DBT and EMDR-only, in adults with PTSD and comorbid (sub)clinical BPD.

## Method

### Objectives

#### Primary objectives

The primary objective of this study is to investigate the clinical efficacy of integrated EMDR-DBT as compared to EMDR-only in adults with PTSD and comorbid (sub)clinical BPD. It is hypothesized that integrated EMDR-DBT will result in a higher effect size (*d* = 1.0) compared to EMDR-only (*d =* 0.5), in clients with PTSD and comorbid (sub)clinical BPD

#### **Secondary objec**t**ives**

The secondary objective of this study is to identify predictors and mediators of the individual treatment response. It is hypothesized that neurobiological factors (i.e. cortisol, FKBP5 and BDNF protein levels and FKBP5 and BDNF methylation status) predict and mediate the individual response to treatment in adults with PTSD and comorbid (sub)clinical BPD. Moreover, this study aims to estimate the cost-effectiveness of integrated EMDR-DBT as compared to EMDR-only. It is hypothesized that, although more expensive in direct medical costs, cost-effectiveness will be higher for integrated EMDR-DBT than for EMDR-only.

### Study design and setting

**Fig. 1:**
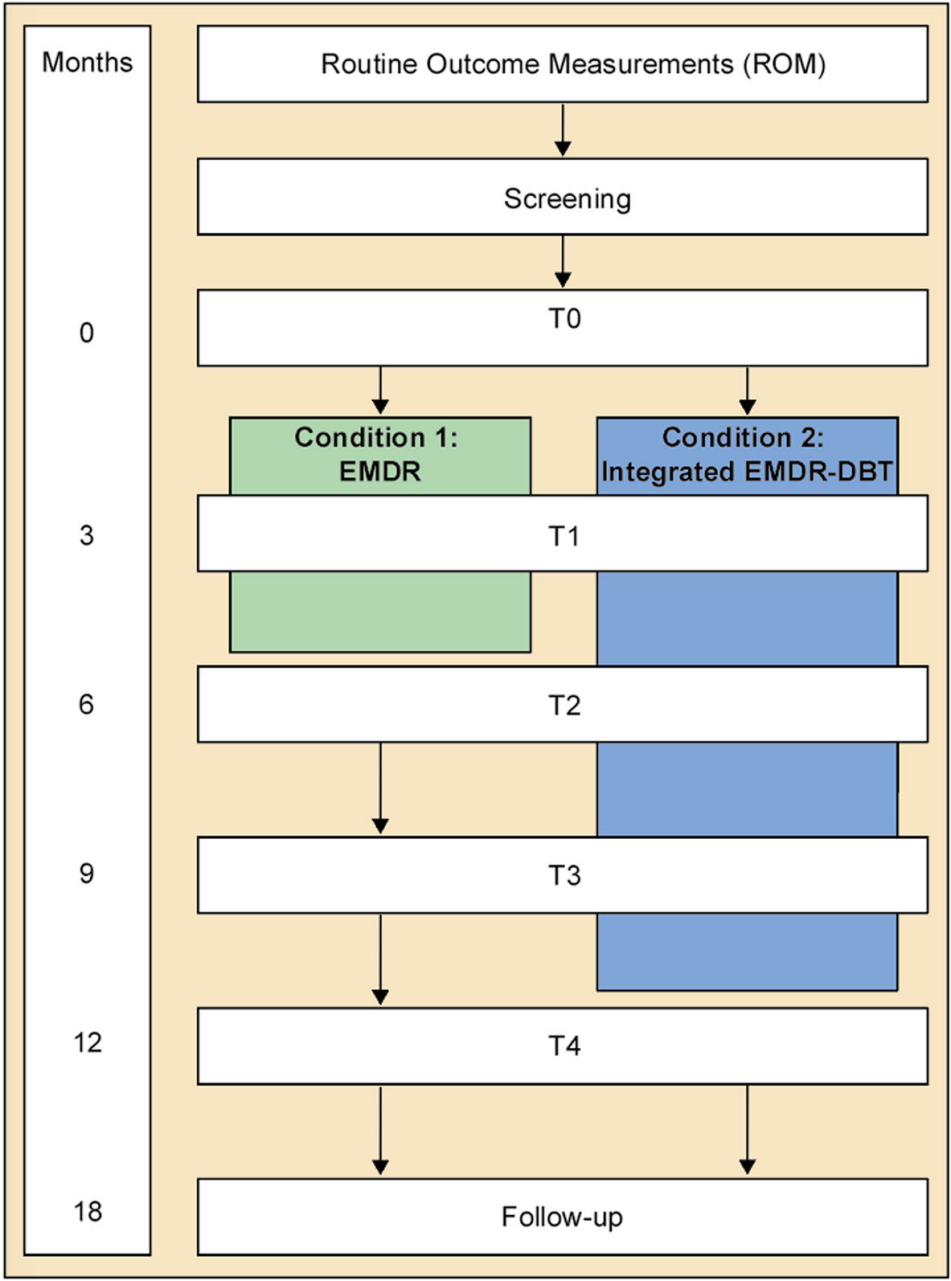
Flow chart for the study design

This study is part of an overarching research project investigating the comparative efficacy of integrated PTSD-PD and PTSD-only treatment, within two parallel randomized controlled trials (for a detailed description of the overarching project, see NCT03833453; for a detailed description of the second randomized controlled trail, see van den End et al. (2009 - under review); for a detailed description of the fMRI substudy, see Aarts et al. (in preparation). The study described herein will follow a randomized controlled design and takes place at two study sites of Sinai Centrum in Amsterdam and Amstelveen, The Netherlands, between June 2017 and June 2022. After a screening visit and obtaining informed consent, clients are randomly assigned to integrated EMDR-DBT or to EMDR-only. Various psychological and biological factors are assessed over a total of six measurement time points (i.e. T0, T1, T2, T3, T4 and a follow-up measure; See Fig. 1 for a flow chart of the study design). A research assistant who is blinded to the intervention conducts the clinical interviews and blood is collected at the hospital. For a detailed overview of the type and timing of measurements, we would like to refer to Table 1.

**Table 1:** Overview of the type and timing of measurements

Measurement	Specification	ROM	Screening	T0	T1	T2	T3	T4	FU
PCL-5	PTSD symptoms	X							
SCID-5-PD screener	PD symptoms	X							
OQ-45	Psychiatric symptoms	X		X	X	X	X	X	X
SCID-5-S	Axis I-disorders		X					X	
SCID-5-PD	PD		X	X				X	
CAPS-5	PTSD			X		X		X	
Demographic questionnaire	Demographics			X					
STAS NL	Anger			X		X		X	
DERS NL	Emotion regulation			X		X		X	
PAI-BOR	BPD symptoms			X		X			X
NSSI	Non-suicidal self-injury			X	X	X	X	X	X
WHODAS 2.0	General functioning			X		X		X	X
EQ-5D-5 L	Quality of life			X		X		X	X
TiC-P	Health care consumption			X		X		X	X
Height/weight				X					
Blood pressure	Systolic and diastolic			X					
Hair sample	HPA-axis (cortisol)			X		X			
BDNF	Protein and methylation			X		X			
FKBP5	Protein and methylation			X		X			

*BDNF* Brain-derived neurotrophic factor, CAPS-5 = Clinician-Administered PTSD scale for DSM-5, *DERS NL* Difficulties in Emotion RegulationScale - Dutch version, *FKBP5* FK506-binding protein, *NSSI*= Non Suicidal Self-Injury screener, *OQ-45* Outcome Questionnaire, *PAI-BOR* Personality Assessment Inventory - Borderline features scale, *PCL-5* PTSD Checklist for DSM-5, *ROM* Routine Outcome Measurement, *SCID-5-PD* Structured Clinical Interview for DSM-5 Personality disorders, *SCID-5-S* Structured Clinical Interview for DSM-5 Syndrome Disorders; *STAS NL* State-Trait Anger Scale – Dutch version; *TiC-P* Trimbos and iMTA questionnaire on Costs associated with Psychiatric illness, *WHODAS* WorldHealth Organization Disability Assessment Schedule 2.0

### Study procedure

#### Participants

##### Recruitment

Study participants are recruited from clients seeking treatment for trauma-related complaints at Sinai Centrum in Amstelveen and Amersfoort, The Netherlands.

For inclusion in the study, clients need to (1) be aged between 18 and 65 years, (2) have sufficient mastery of the Dutch language, (3) be diagnosed with PTSD according to the DSM-5, (4) meet at least four criteria of BPD according to the DSM-5.

Clients are excluded from study participation in case of (1) current psychosis, (2) primary diagnosis of paranoid, schizoid, schizotypal, narcissistic, histrionic or antisocial personality disorder, (3) mental retardation (i.e. IQ < 70), (4) comorbidity interfering with treatment or randomisation (i.e. severe outward aggression, current addiction disorder without the capacity or intention to stop for treatment, eating disorders with BMI < 17), and/or (5) participation in another research project that requires the patient to receive psychotherapy outside of the current study. In case of acute suicidality, recent suicide attempts and/or non-suicidal self-injury, the possibility to start (or to continue) treatment is assessed by a clinician on an individual basis. If necessary, the treatment is adjusted according to the clients’ needs. For instance, the patient can create a patient safety plan together with a therapist aimed at mentally preparing the patient for participation in the study. In addition, a psychiatrist can be involved who may or may not decide to medically treat the patient. A social worker or a psychiatric nurse can also be involved in order to provide extra support to the patient. As long as no additional psychological intervention is added, the patient remains within the study. If these options deem insufficient (i.e. the patient remains actively self-harming or at acute risk for suicide) the therapist may decide to intensify the EMDR treatment by offering multiple sessions per week within the outpatient setting, or to continue treatment within a clinical setting. In the latter two cases, the patient is excluded from the current study and considered a dropout. In case of high dose benzodiazepine use (i.e. equivalent to 3 × 10 mg oxazepam on a daily basis) which may inhibit the response to EMDR treatment, patients are required to be abstinent on the day of the EMDR session and one day before and after the EMDR session. Additionally, patients using psychotropic medication need to have a stable medication regimen for at least three weeks prior to admission to the study. All treating psychiatrists are aware of these medication guidelines and monitor the patients closely. Psychiatrists discuss any necessary deviations from the study protocol with the researchers. In addition, medication use is monitored by means of self-report (i.e. see Methods; TiC-P). After treatment completion each participant’s medication record is examined by one of the researchers in order to record all deviations for later analyses.

##### Sample size calculation

**Fig. 2:**
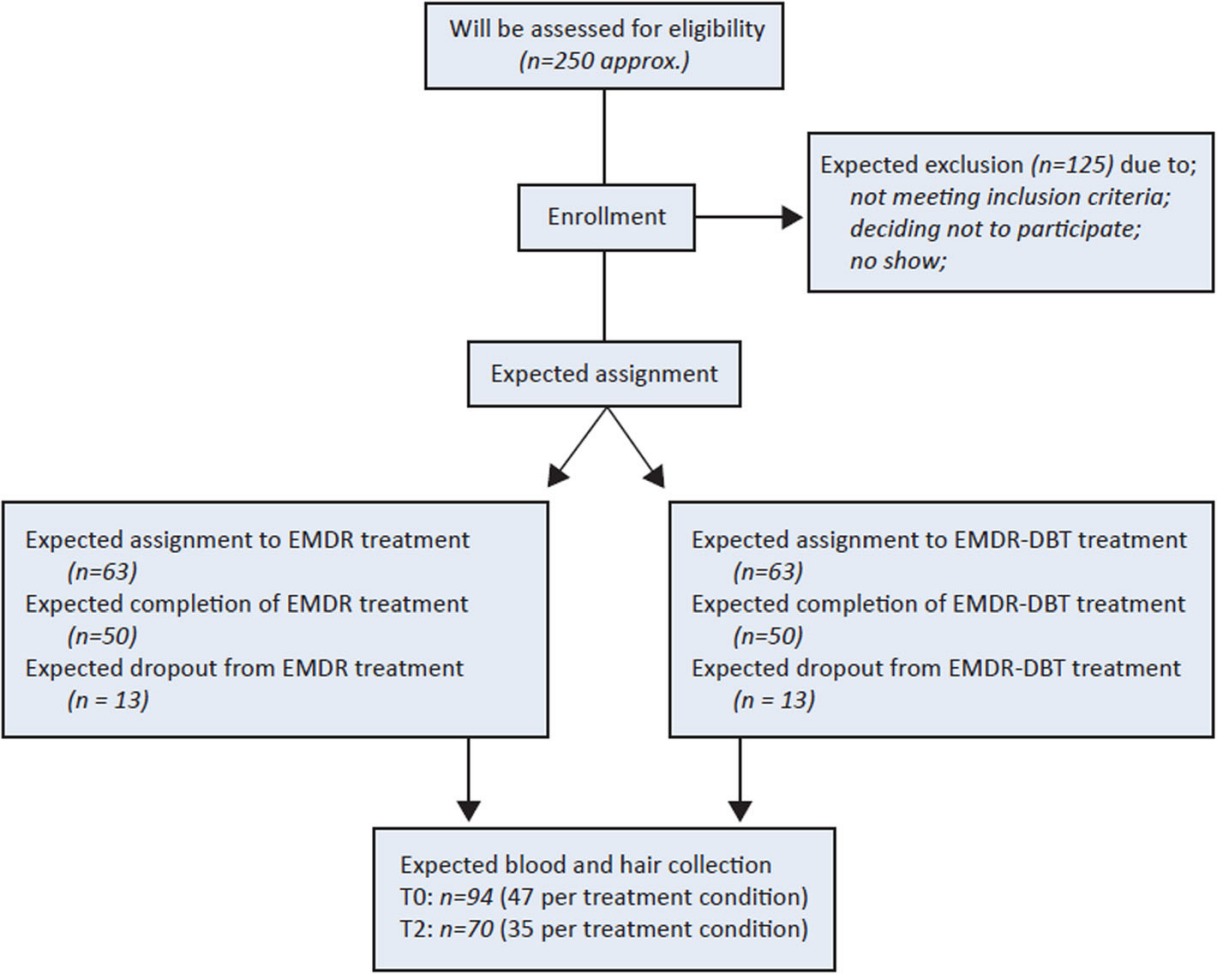
Flow diagram of the expected study progress throughout enrolment and assignment to the interventions

A power calculation was conducted based on Twisk [52], with a two-sided .05 significance level and a statistical power of 80%. A small Cohen’s’ d effect size (i.e. d ≤ 0.5) was estimated for the EMDR-only condition and a large Cohen’s d effect size (i.e. d ≥ 1.0) was estimated for the integrated EMDR-DBT condition, in patients with PTSD and (sub)clinical BPD [11, 28]. To detect a minimal clinical relevant difference of 0.5 standard deviations on the CAPS-5 with two follow-up measurements and an intrapersonal correlation coefficient of 0.5, a sample size of 50 patients is required for each treatment condition. Taking into account a dropout rate of approximately 25%, 63 clients per treatment condition will be included [53, 54]. The expected participant selection process is illustrated in Fig. 2.

#### Assessments

Clients are assessed at a screening appointment and during the first week of pre-treatment admission (T0), as well as three months after the start of treatment (T1), directly after completion of the EMDR treatment (T2), and nine (T3), twelve (T4) and eighteen months (FU) after the T2 measurement (see Fig. 1). A doctoral-level clinician who received initial accredited training (i.e. Arq Academy, Diemen, Netherlands) will administer the clinical interviews. According to the preference of the client, online questionnaires are filled in at home or at Sinai Centrum. A detailed overview of the type, duration and timing of all measurements is given in Table 1.

##### Screening

As part of the standard care, all clients applying for treatment at Sinai Centrum are asked to complete a series of online questionnaires during the first week of pre-treatment admission (i.e. Routine Outcome Measurements (ROM); See Table 1). If the psychologist who performs the intake deems the client eligible based on the aforementioned in- and exclusion criteria and if the client meets at least four BPD criteria on the SCID-5-PD screener, the client receives an information folder of the study. In case of interest, the psychologist informs one of the researchers, who in turn contacts the client by phone to further explain the study procedure. If the client wishes to participate, the client is invited to Sinai Centrum for a SCID-5-S and SCID-5-PD interview of approximately 120 min conducted by the researcher. If the client meets at least four BPD criteria on the SCID-5-PD, the client is allowed one week time to reflect on the potential benefits and risks and to decide upon participation in the study. The client is then once again asked whether he or she wants to participate in the study. If the client still wishes to participate, an appointment for randomisation and obtaining informed consent is scheduled.

##### Informed consent, randomisation and blinding

After explanation of the aims, methods, benefits and potential hazards of the study, the investigator obtains written informed consent from each client participating in the study. Before random assignment, clients are told that they have a 50% change of being assigned to either treatment condition. Next, an independent researcher pulls an envelope from an ordered stack of numbered envelopes to determine the treatment condition. Randomisation will be based on block randomisation (*n* = 4 per block), to guarantee a balance between conditions over time. Neither researchers, therapists nor study participants can be blinded to treatment condition given the nature of the interventions. However, blinding is partly maintained by ensuring that clients are blinded to research hypotheses and by ensuring that the research assistant who performs the measurements is masked to the client’s treatment condition, hereafter referred to as independent evaluator. Last of all, all clients receive a gift certificate of €10 per measurement.

##### T0

On arrival at the baseline appointment, an independent evaluator measures height, weight and resting heart rate and blood pressure. The independent evaluator then collects a sample of hair and administers a questionnaire regarding certain hair characteristics (i.e. natural hair colour, bleaching, dying, perming and the use of hair products and corticosteroids). Blood is collected at the hospital. The CAPS-5 interview is then administered.

A subgroup (approximately 25%) of all patients will be asked to participate in fMRI research before and after EMDR treatment. In this subgroup, structural and functional MRI, with resting-state and emotion processing (face recognition) will be performed. In addition, working memory and response inhibition are assessed in all patients at T0, through the computer based N-Back and the Stop-Signal Task respectively. The fMRI study is not part of the study outlined in this design paper and will therefore not be further described (for a more detailed description of the fMRI study, see NCT03833531.

##### T1, T3 and follow-up

At T1 and T3, clients fill in online questionnaires with a personalized login code (See Table 1).

##### T2

At T2, the client is invited to Sinai Centrum for a CAPS-5 interview by an independent evaluator. Hair and blood samples are collected from those clients who also gave these samples at baseline. Lastly, clients fill in online questionnaires (See Table 1).

##### T4

At T4, the client is invited to Sinai Centrum for a SCID-5-PD and CAPS-5 interview conducted by an independent evaluator. Clients also fill in online questionnaires (See Table 1).

### Data collection and management

#### Measurements

##### PTSD checklist for DSM-5

The PTSD Checklist for DSM-5 (PCL-5) is a 20-item self-report measure that assesses the presence and severity of the *DSM-5* symptoms of PTSD [55, 56]. The PCL-5 includes a relatively short criterion A section and the severity of criterion B to E symptoms is scored on a five-point rating scale, ranging from not at all (0) to extremely (4). The PCL-5 has strong internal consistency, strong test-retest reliability and strong convergent and discriminant validity [57].

##### Clinician-administered PTSD scale for DSM-5

The Clinician-Administered PTSD Scale for DSM-5 (CAPS-5) is a structured diagnostic interview to assess the frequency and severity of DSM-5 PTSD symptoms [55, 56]. The interview consists of 30 items (e.g. *“In the past month, have you had any unwanted memories of (EVENT) while you were awake, so not counting dreams?”*). Symptoms in relation to the past month are examined, with a severity score of two or higher indicating the presence of a symptom. In addition to symptom severity, CAPS-5 provides information about the onset and duration of the symptoms, as well as the dissociative subtype, response validity and symptom changes compared to a previous CAPS-5 measurement. The CAPS-5 total severity score has high internal consistency and inter-rater reliability, and good test-retest reliability. In addition, CAPS-5 diagnosis has strong inter-rater reliability and test-retest reliability, as well as strong correspondence with PTSD diagnoses based on the CAPS for DSM-4 [58]. Each assessor will receive an accredited training from ARQ Academy (Diemen, Netherlands) to administer the interview.

##### Structured clinical interview for DSM-5 personality disorders

The Structured Clinical Interview for DSM-5 Personality Disorders (SCID-5-PD) is a semi-structured interview to assess the presence and severity of the DSM-5 personality disorders [59]. Diagnoses are made either categorically (present or absent) or dimensionally (summing the ratings for each symptom). Although the fifth version of the SCID-PD has not been validated yet, its predecessor SCID-II-PD showed excellent inter-rater reliability, fair to good test-retest reliability and satisfactory internal consistency [60–62]. Moreover, a pilot study demonstrated an excellent inter-rated of the Italian translation of the SCID-5-PD as well [63]. Each assessor will receive an accredited training from ARQ Academy (Diemen, Netherlands) to administer the interview.

##### Structured clinical interview for DSM-5 syndrome disorders – Dutch version

The Structured Clinical Interview for DSM-5 Syndrome Disorders (SCID-5-S) is a semi-structured interview to assess the presence and severity of the DSM-5 disorders most commonly seen in clinical settings ([64]; American Psychiatric Associaction [65]. Specifically, the following disorders are assessed: major depressive disorder (current) with or without (hypo)manic episodes (current); psychotic and related symptoms (delusions, hallucinations); alcohol use disorder (current); substance use disorder (current); panic disorder (current); agoraphobia (current); social anxiety disorder (current); generalized anxiety disorder (current); obsessive-compulsive disorder (current); anorexia nervosa (current) and bulimia nervosa (current). The SCID-5-S has acceptable to excellent internal consistency and fair to substantial test-retest reliability [66].

##### Demographic questionnaire

The Demographic questionnaire assesses basic personal, social and medical data: 1) gender; 2) age; 3) marital status; 4) daily housing/living situation; 5) number of children and their age; 6) ethnic background; 7) highest level of achievement in education; 7) current work and education; 8) religion; 9) duration of trauma-related complaints; 10) previous individual and group treatments; 11) previous hospitalization; 12) substance abuse or dependence.

##### Outcome questionnaire 45

The Outcome Questionnaire 45 (OQ-45) is a 45-item questionnaire to measure clinical outcome [67]. Each item is scored on a five-point rating scale, ranging from never (0) to almost always (4). The symptom distress (SD) subscale has 25 items that assess the most common disorders in public mental health (i.e. depression, anxiety and addiction). The Interpersonal Relations (IR) subscale contains 11 items that assess relational functioning. The Social Role (SR) subscale contains nine items assessing functioning in school, work and leisure. The Dutch version of the OQ-45 has good criterion validity, sufficient reliability, adequate concurrent validity and is highly sensitive to change [68].

##### Personality assessment inventory-borderline features scale

The Personality Assessment Inventory-Borderline Features (PAI-BOR) Scale is a self-report measure assessing the presence and severity of BPD [69]. The BAI-BOR consists of four subscales of six items each, reflecting four main characteristics of BPD: affective instability, negative relationships, identity problems and self-harm. Each items is rated on a four-point scale, ranging from false (0) to very true (3). A total PAI-BOR score of 38 or more indicates the presence of significant BPD features, whereas a score of 60 or more indicates typical borderline personality functioning. The Dutch version of the PAI-BOR has good internal consistency, satisfactory reliability and acceptable test-retest reliability [70].

##### Difficulties in emotion regulation scale

The Difficulties in Emotion Regulation Scale (DERS) is a 41-item self-report measure to assess clinically relevant difficulties in emotion regulation [71]. DERS items reflect difficulties within four dimensions of emotion regulation: awareness and understanding of emotions, acceptance of emotions, the ability to engage in goal-directed behaviour and to refrain from impulsive behaviour, and access to effective emotion regulation strategies. Items are scored on a five-point scale, ranging from almost never (1) to almost always (5). Although the Dutch version of DERS (DERS-NL) has not been validated yet, the original DERS showed high internal consistency, good test-retest reliability and adequate construct and predictive validity [71].

##### Zelf-analyse Vragenlijst

The Zelf-analyse Vragenlijst (ZAV) is a Dutch version of the original State Trait Anger Scale (STAS [72, 73];). The ZAV assesses the intensity of state and trait anger through 20 self-report items, ranging from 1 (almost never) to (4) always. For the study described herein, only trait anger is assessed using the 10-item subscale of the ZAV. The STAS has acceptable to strong internal consistency and psychometric properties for the ZAV trait anger subscale are adequate [73, 74].

##### Non-suicidal self-injury screener

The Non-suicidal self-injury (NSSI) screener consists of 7 multiple-choice items assessing non-suicidal self-injury [75]. In case of an affirmative responses to the item *‘Have you ever done any of the following with the purpose of intentionally hurting yourself?’* engagement in NSSI is determined. The use of such a single, dichotomous item for assessing NSSI is common in NSSI research and renders consistent prevalence estimates [76].

##### Trimbos/iMTA questionnaire for costs associated with psychiatric illness

The Trimbos/iMTA questionnaire for costs associated with psychiatric illness (TiC-P) is a self-report questionnaire assessing direct medical costs and productivity costs due to absence from work or reduced efficiency during work in patients with a mental disorder [77]. The first part of the TiC-P includes 14 structured yes or no questions on the use of medical resources, each followed by a question on the volume of medical consumption. The second part includes five items on work absence, reduced efficiency at work and related productivity losses. The TiC-P showed perfect to moderate inter-rater reliability, satisfactory construct validity and satisfactory test-retest reliability in a clinical sample of patients with mood and anxiety disorders [78].

##### EuroQol five-dimensional five-level (EQ-5D-5 L) questionnaire

The EQ-5D-5 L is a self-report questionnaire assessing health-related quality of life [79]. The EQ-5D-5 L contains two components: health state description and health-state evaluation. In the description part, health state is measured through five dimensions: mobility, self-care, usual activities, pain and discomfort, and anxiety and depression. Each dimension is scored on a five-level scale, ranging from no problems (1) to extreme problems (5). Responses to the five dimensions are weighted and summed to create a total score between 0 (i.e. deceased) and 1 (i.e. full-health). In the evaluation part, the client is asked to mark his or her current health status on a 20 cm Visual Analogue Scale (VAS) with end points of 0 (i.e. the worst health you can imagine) and 100 (i.e. the best health you can imagine). The EQ-5D-5 L has good reliability and good convergent validity with the World Health Organization 5-item Well Being Questionnaire [80, 81].

##### World Health Organization disability assessment schedule 2.0 (WHODAS 2.0)

WHODAS 2.0 is a 36-item self-report questionnaire assessing the daily function of activity and participation within the 30 previous days, including the following six domains: Cognition, Mobility, Self-care, Getting along, Life activities and Participation [82]. The responses on each item range from no difficulty (1) to extreme difficulty (5). Responses to the six dimensions are weighted and summed to create a total score between 0 (no disability) and 100 (complete disability). The WHODAS 2.0 has excellent internal consistency and test-retest reliability. In addition, face, concurrent and construct validity of WHODAS 2.0 are supported [82].

##### Body measures (height, weight)

Height is measured using a Harpenden stadiometer, consisting of a vertical backboard with a weighted horizontal cursor fixed to it at 90 degrees. A mechanical counter running on track is placed on the head, after which the height can be read from the analogue backboard. The stadiometer technique has an excellent intra- and inter-rater reliability [83].

Weight is measured using the scale technique, which provides a quantative outcome (kilogram) of the amount of weight in standing position. The client is instructed to stand upright on a standard analogue scale ranging from zero to approximately 150 kg. The analogue scale technique has an accuracy of ±0.45 kg [84].

##### Hair cortisol

Hair cortisol provides a retrospective long-term, month-by-month measure of systematic cortisol exposure [41]. A hair sample will be collected by cutting the most proximal one centimetre segment of the vertex posterior of the head, thereby minimizing intra-individual variations in cortisol concentrations [41]. Given that hair has an overall growth rate of 1 cm per month, the most proximal one centimetre segment to the scalp constitutes last month’s cortisol production. The second most proximal segment to the scalp constitutes the second to last month’s cortisol production and so forth. After hair collection, the researcher administers a questionnaire regarding certain hair characteristics (i.e. natural hair color, bleaching, dying, perming and the use of hair products and corticosteroids). Cortisol levels might be slightly influenced by hair treatment, but remain unaffected by the use of hair products, gender or age [85]. Hair samples are marked with a subject number as described in section *Declartions – Availability of data and materials* below, and are stored at room temperature for future analysis of hair cortisol.

##### Fasting blood sample

Fasting blood is collected at Amstelland Hospital in Amstelveen and Meander Medical Centre in Amersfoort, the Netherlands. Serum and EDTA vacutainers with a volume of 6 ml (Becton, Dickinson and Company, Franklin Lakes, NJ) are centrifuged within one hour upon blood draw and divided into five aliquots of 500 μl each. Serum and plasma samples are labelled with the correct assessment and a unique subject number as described in section *Declartions – Availability of data and materials* below, and stored at − 80 °C for future assays. A third vacutainer with a volume of 4 ml is sent to the department of clinical genetics at the Amsterdam University Medical Center for DNA-isolation. DNA is isolated within 5 days upon blood draw, using a method based on magnetic beads with the Chemagen chemagic MSM1 instrument (Chemagen, Baesweiler, Germany), which was replaced by the Qiasymphony instrument (Qiagen, Hilden, Germany) since January 1st, 2019. DNA samples are stored at − 20 °C for future epigenetic analyses.

##### Heart rate and blood pressure

Heart rate and blood pressure are reliable measures of sympathetic and parasympathetic autonomic nervous system functioning [86, 87]. Clients are instructed to sit still in a seated position, during which resting heart rate and blood pressure are measured using a digital upper arm sphygmomanometer connected to the non-dominant arm.

##### Treatment compliance

EMDR treatment compliance is operationalized as completion of the treatment within a maximum of 20 weeks. DBT treatment compliance is operationalized as less than 25% missed group sessions, including no-shows and cancelled appointments.

##### Therapist adherence

In order to measure therapists’ adherence to the EMDR protocol, psychotherapists videotape each treatment session. Out of all treatment sessions, a total of 10% randomly chosen videotaped sessions will be independently rated using the EMDR Fidelity Scale [88]. The EMDR Fidelity Rating Scale (EFRS) ranges from 0 (no adherence) to 3 (very good adherence), with a cut-off score of 2 for acceptable fidelity.

To establish adherence to the DBT protocol, psychotherapists audio-record each individual treatment sessions. Of all individual treatment sessions, a total of 10% randomly chosen audio-recorded sessions will be independently rated using the DBT Adherence Coding Scale [89]. The DBT Adherence Coding Scale consists of 63 items, providing an overall score ranging from 0 to 5. A rating of 4 reflects good DBT adherence.

### Interventions

#### Eye movement desensitization and reprocessing (EMDR)

The EMDR treatments are performed by certified EMDR therapists who received accredited training. Therapists receive biweekly face-to-face supervision with coequal co-workers. The aim of the supervision is to review cases and to discuss adherence to the treatment protocol, in order to maintain quality and homogeneity of the intervention. In addition, treatment progress is evaluated within a weekly multidisciplinary consultation team.

A minimum of 12 and a maximum of 18 weekly EMDR sessions are conducted. Each EMDR session has a duration of 60 min. The entire treatment consists of the eight phases described below.

During session 1 the focus lies on history taking and case formulation. Trauma-related complaints are mapped out and possible targets for EMDR are identified using the LEC-5. The therapist explains the theoretical background for EMDR and describes the actual steps in the process. In consultation with the client, the therapist develops a treatment plan for the following sessions.

During session 2 the traumatic memory is identified. In case of multiple traumatic memories, the most distressing traumatic memory is selected as the target memory. Next, a negative cognition (NC) about the self that is associated with the target trauma and a replacing positive cognition (PC) about the self is selected. The client assesses the validity of the positive cognition on a seven-point scale (Validity of Cognition (VoC) scale 1–7). The client then reports the physical tension associated with the target event and scales the disturbance level on an eleven-point scale (i.e. Subjective Units of Disturbance (SUD) scale 0–10). Next, the desensitization phase starts. The client is instructed to hold in focus a picture of the most disturbing traumatic event, along with the associated negative self-perception and physical tension. The client simultaneously engages in the processing of a bilateral stimulus (BLS), consisting of rapid hand movements. After each set of BLS, the client is asked to comment on whatever comes to awareness. When the client reports no distress related to the targeted trauma memory (i.e. SUD equals zero) *and* when the PC is experienced as being totally valid (i.e. VOC equals 7), session 2 is completed. If the target memory was not fully processed during this session (i.e. if SUD does not equal 0), the therapist provides instructions and techniques to the client that promote containment and ensure safety until the next session. The client is instructed to keep record of new sensations and/or experiences, and to report these at the beginning of the next session.

At the beginning of session 3 until the second to last session, the SUD and the PC from the previous session are revisited, reconfirmed and the validity of the PC is scaled on the VoC again. Sets of BLS are applied until the SUD equals zero and the PC is experienced as being totally valid. Depending on the client’s reports, the therapist chooses the next target memory and its associated thoughts and sensations (i.e. NG, PC, VoC and SUD), after which the desensitization phase is repeated as described in session 2.

During the last session, the therapist re-evaluates whether treatment effects have maintained and checks if there are still any target-related memories or sensations that demand attention. The client is instructed to mentally scan his or her body to check whether there are still any target-related sensations or tensions. If there are, the therapist continues BLS until negative sensations subside or until positive sensations are fully experienced. If the SUD does not equal zero or if the VoC does not equal 7, the cycle begins again at the desensitization phase as described in session 2.

#### Dialectical behaviour therapy (DBT)

Dialectical Behaviour Therapy (DBT) is a cognitive behavioural therapy used to treat individuals with BPD. Its main focus lies on teaching the client how to cope with dysregulated emotions and how to improve interpersonal relationships. For the current study, an additional objective of DBT is to reduce BPD symptoms such that clients can simultaneously receive EMDR treatment. All patients who are randomised to integrated EMDR-DBT continue to concurrently receive all modes of DBT (i.e. individual psychotherapy, group skills training and phone coaching; further described below) during their EMDR treatment.

DBT treatments are performed by certified DBT therapists who have completed at least the three-day introductory training in DBT principles administered by Dialexis (i.e. the training institute of the Dutch DBT association, see https://www.dialexisadvies.nl/). To ensure adherence to the DBT strategies, therapists will receive biweekly supervision within a consultation group operating according to the DBT principles [90]. In addition, there will be 3-h external supervision once in every three months. In order to ensure treatment confidentiality, the DBT therapist is never the same person as the EMDR therapist.

DBT has an overall treatment length of 54 weeks. The treatment starts with six weekly individual DBT sessions of 45 min each. During this pre-treatment phase, the therapist explains the theoretical background for DBT. In consultation with the client, the therapist develops an individualized treatment plan for the following sessions. The client is prepared for group participation by determining personalized treatment goals of which the client keeps a daily record on so-called diary cards. The diary cards provide a guideline for the individual therapy and must therefore be completed prior to each individual session. During the pre-treatment phase, a crisis plan on how to deal with daily life crises is made as well.

After the pre-treatment phase, clients receive a combination of individual psychotherapy and group skills training. Individual DBT therapy sessions have a duration of 45 min each and take place every other week on a day when there is no group session. As already mentioned, the client’s personal diary cards provide the guideline for the individual therapy sessions. During these sessions, behavioural skills that are learned during the group sessions are adapted to the client’s personal life. After the sixth group skills training session, the weekly EMDR sessions commence.

The group skills training consists of 48 weekly group sessions of 150 min each, including a 15-min break. Each group consists of a maximum of 10 clients led by two therapists. The group leader makes a video recording of each session. There are four main types of skills that are covered in the group skills training. First of all, mindfulness-related skills help the client focus on the present and care for oneself in the moment. Secondly, interpersonal effectiveness skills teach the client how to obtain one’s needs while maintaining a healthy relationship, how to be assertive in solving interpersonal problems and how to decrease social isolation. Thirdly, the client is taught emotion regulation skills such as identifying and labelling emotions, inhibiting inappropriate behaviour related to strong emotions, and increasing positive emotions. Last of all, the distress tolerance module includes crisis survival skills aimed at making healthy decisions instead of destructive emotional decisions and reality acceptance skills. Weekly homework is assigned in order to encourage generalization of skills taught during the group sessions.

Lastly, the client can call his or her therapist outside of the therapy to receive guidance with difficult at-the-moment situations, referred to as ‘phone coaching’. Phone coaching takes place when crisis behaviour has not yet taken place, when there is a willingness to practice new skills and when there is a crisis. The purpose of phone coaching is to reduce suicidal behaviour, to increase generalization of skills and to reduce feelings of conflict or alienation from the therapist.

### Data analysis

The statistical analyses of the primary outcomes will be performed on an intention-to-treat basis. All significance levels will be set at 0.05, with two-sided hypothesis testing. Pre-treatment group differences are assessed using independent samples t tests for continuous data and χ^2^ test for categorical data. Missing data will be replaced using methods such as multiple imputations, assuming that the data will be missing at random [91].

#### Treatment effects

A random intercepts and slopes multilevel model will be used to examine the comparative efficacy of the EMDR-DBT and EMDR-only treatment, in which the measurement time points are nested within clients. At level 1 of the model, the primary outcome measures (*Yijb;* scores on the CAPS-5 and SCID-5-PD) vary *within* clients over time (*X1ijb*) as a function of client-specific growth curves. At level 2 of the multilevel model, the client-specific change parameters vary randomly *between* clients as a function of the client’s treatment condition (*X2jb*). The combination of the level 1 and level 2 models results in a mixed linear model with fixed and random coefficients. Fixed coefficients include an intercept (*y00b*; scores on primary outcome measures if time and treatment condition are set to zero) and a linear or quadratic slope (*y10b;* effect of time on primary outcome measures). Quadratic slopes possibly fit the data better than linear slopes, because treatment efficacy is generally a curvilinear function of time (e.g. response to treatment decreases over time). For each client, random coefficients are allocated to the client-level intercepts (*U0jb;* client-specific deviations from group’s predicted intercepts) and slopes (*U1jb;* client-specific deviations from group’s predicted slopes). Lastly, the interaction between time and treatment is modelled (*y11b*). A cAR(1) structure will be used to model the residual covariance matrix. Responders will be defined as clients with a post-treatment score of at least a half standard deviation below the pre-treatment score.

$$ Level\ 1:\kern3.75em Yijb=\beta 0 jb+\beta 1 jb\ast X1 ijb+\varepsilon ijb $$

$$ {\displaystyle \begin{array}{l} Level\ 2:\kern3.75em \beta 0 jb=\gamma 00b+\gamma 01b\ast X2 jb+U0 jb\\ {}\begin{array}{c}\beta 1 jb=\gamma 10b+\gamma 11b\ast X2 jb+U1 jb\\ {}\end{array}\\ {}\kern0em \end{array}} $$

The described random-effects multilevel model provides accurate statistical inferences for nested data within a hierarchical linear model (HLM) structure. By including random intercepts and slopes, within-subject correlations can be modelled, thereby minimizing the chance of underestimating the model’s variance and falsely rejecting the null hypothesis (i.e. Type I error).

### Cost-utility and cost-effectiveness

Both the integrated EMDR-DBT and EMDR-only will be economically evaluated alongside the randomised trial and will be performed according to the intention-to-treat principle. We will take into account the CHEERS statement [92] and the 2015 ISPOR good research practices task force report on cost-effectiveness analysis alongside clinical trials [93]. In brief, cost-effectiveness will be performed taking the societal perspective in the base case scenario, in order to evaluate whether EMDR-DBT is cost-effective compared to EMDR-only. Hence, we will take in account at least the following costs: the cost of offering EMDR-DBT or EMDR-only, costs of other healthcare uptake besides EMDR(−DBT) and costs of productivity losses due to absenteeism or presenteeism. Health care costs will be valued based on standard Dutch cost prices (Zorginstituut [94]). Costs of productivity losses will be based on the gender- and age-specific labour costs. To facilitate comparison of our findings with previous economic evaluations in psychiatric research, healthcare costs will be assessed using the Tic-P, treatment effects will be based on the CAPS-5, and Quality Adjusted Life Years (QALYs) will be calculated using the area under the curve method, using the responses to the EQ-5D-5 L. The time horizon of the economic evaluation will be 18 months post-randomisation. Together, these measures will be re-calculated into incremental cost-effectiveness ratios (ICERs), defined as the difference in mean costs divided by the difference in mean effects of the two interventions [95]. Secondly, cost-utility will be assessed in order to evaluate whether EMDR-DBT leads to more improvement in health-related quality of life per euro spent than EMDR-only. Health care costs and QALYs will be re-calculated to obtain incremental cost-utility ratios (ICURs), defined as the difference in mean costs divided by the difference in QALYs gained by the two interventions [96]. To estimate stochastic uncertainty around the ICERs/ICURs, the bootstrap approach will be used. Bootstrapped data will also be used to plot cost effectiveness acceptability curves. One-way sensitivity analyses and/or scenario analyses will be performed to evaluate the robustness of our findings against misspecifications of key cost drivers.

### Prediction effects

It is hypothesized that individual treatment outcome will be predicted by levels of hair cortisol, FKBP5 and BDNF protein levels and FKBP5 and BDNF methylation status. The predictive values of these variables will be investigated using regression analyses with their mean change scores from pre- to post-treatment and pre-treatment to follow-up as the dependent variables and treatment condition as the independent variable (EMDR, EMDR-DBT). Responders will be defined as clients with a post-treatment score of at least a half standard deviation below the pre-treatment score.

### Mediation effects

**Fig. 3:**
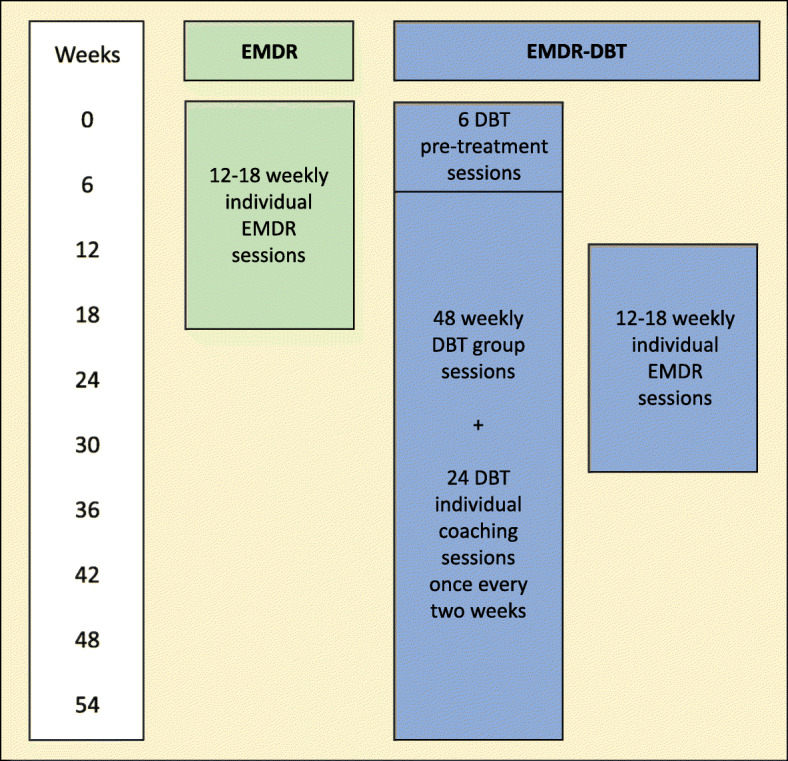
Treatment outline per week for the EMDR-only condition and the EMDR-DBT condition

**Fig. 4:**
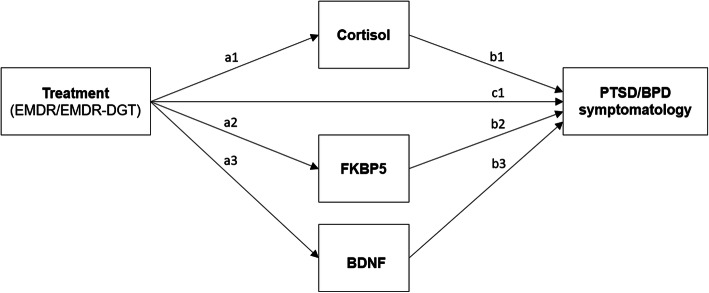
Relationship between treatment condition (i.e. EMDR, EMDR-DBT) and primary outcome measures (i.e. post-treatment scores on the CAPS-5 and SCID-5-PD), proposedly mediated by pre-to post-stressor changes in levels of cortisol, FKBP5 and BDNF protein levels and FKBP5 and BDNF methylation status. It is hypothesized that the regression of the primary outcome measures on treatment condition, ignoring the mediators, will be significant (c1). Secondly, the regression of the mediators cortisol, FKBP5 and BDNF protein levels and FKBP5 and BDNF methylation status on treatment condition are hypothesized to be significant as well (a1, a2 and a3 resp.). In addition, the regression of the primary outcome measures on the mediators are also expected to be significant (b1, b2 and b3 resp.). Lastly, it is hypothesized that, when controlling for the mediators, treatment condition will significantly predict the scores on the CAPS-5 and SCID-5-PD, supporting partial mediation

The effect of time on the primary outcome measures is expected to be partially mediated by pre- to post-treatment changes in levels of hair cortisol, FKBP5 and BDNF protein levels and FKBP5 and BDNF methylation status. A mediation analysis will be conducted to estimate direct (i.e. Figure 3 - path *c1*) and indirect paths (i.e. Figure 3 - paths *a1, a2, a3, b1, b2 and b3*) of casual influence from treatment condition (EMDR, EMDR-DBT) to primary outcome measures, through the proposed mediators. In order to calculate the direct and indirect effects of this parallel multiple mediation model, Model 4 in the PROCESS macro of Hayes (2013) will be used. The independent variable will be dummy-coded to include the EMDR-only condition (0) and the EMDR-DBT condition (1). Change scores of the mediators and the outcome variables will be used, in order to preserve the expected within-between interaction. The parallel multiple mediation model is shown graphically in Fig. 4.

It is hypothesized that the regression of the CAPS-5 and SCID-5-PD scores on treatment condition (i.e. EMDR, EMDR-DBT), ignoring the mediators, will be significant. Secondly, the regressions of the mediators cortisol, FKBP5 and BDNF protein levels and FKBP5 and BDNF methylation status on treatment condition are hypothesized to be significant as well. In addition, the regression of the CAPS-5 and SCID-5-PD scores on the mediators are also expected to be significant. Lastly, it is hypothesized that, when controlling for the mediators, treatment condition will significantly predict the scores on the CAPS-5 and SCID-5-PD, supporting partial mediation.

## Discussion

The study described in this design paper constitutes the first randomised controlled trial investigating the comparative clinical efficacy and cost-effectiveness of integrated EMDR-DBT and EMDR-only in patients with PTSD and comorbid (sub)clinical BPD. It is hypothesized that integrated EMDR-DBT will result in a higher effect size and a higher response rate than EMDR-only. Moreover, it is hypothesized that, although more expensive, cost-effectiveness will be higher for integrated EMDR-DBT than for EMDR-only treatment. The secondary objective of this study is to identify predictors and mediators of the individual treatment response. It is hypothesized that neurobiological variables (hair cortisol, FKBP5 and BDNF protein levels and FKBP5 and BDNF methylation status) predict and mediate the individual response to treatment in adults with PTSD and comorbid (sub)clinical BPD.

This study has several strengths, including being the first to investigate the comparative efficacy of integrated EMDR-DBT and EMDR-only in patients with PTSD and comorbid (sub)clinical BPD. The inclusion of candidate predictors and mediators of the response to treatment, which constitute a necessary prelude to optimizing and individualizing treatment, further increases the clinical meaningfulness of the results. Results will reveal which treatment works best for which individual patient, thereby guiding and optimizing treatment choices while minimizing exclusion and dropout rates. For instance, treatment type and duration could be specified by the biological layout of the patient in question. Moreover, scientific rigor is emphasized through the use of a randomized design, a large sample with adequate statistical power to detect clinically meaningful effects, validated outcome measures in both psychological and physiological domains and a follow-up assessment. Last of all, findings will most likely generalize to other patients with PTSD and comorbid (sub)clinical BPD, as this study is conducted in a common clinical setting at two study sites with a relatively heterogeneous patient population in terms of age, gender, ethnicity and type of trauma.

While the strengths of this study design are encouraging, findings should be interpreted in the light of certain limitations. The first limitation of the present study is the lack of a non-active control group, such as a waiting-list group. The presence of a non-active control group would allow for the assessment of non-specific factors that might influence the dependent variables, such as the passage of time. After all, the hypothesized improvements in PTSD and (sub)clinical BPD could be caused by the passage of time rather than by the interventions. However, the fact that both EMDR and DBT constitute widely used, evidence-based interventions for PTSD and BPD does not support this view. Moreover, according to Paragraph 29 of the Declaration of Helsinki, it would be unethical to withhold evidence-based therapies from patients: *“The benefits, risks, burdens and effectiveness of a new method should be tested against those of the best current prophylactic, diagnostic, and therapeutic methods. The World Medical Association hereby reaffirms its position that extreme care must be taken in making use of a placebo-controlled trial and that in general this methodology should only be used in the absence of existing proven therapy”* [97]. For these reasons, it was decided not to include a waiting-list group in the current study. A second limitation could be the considerably shorter treatment duration of EMDR-only as compared to the integrated EMDR-DBT. Given that the EMDR treatment is more than twice as short as the integrated EMDR-DBT, recovery might be faster in the short-term EMDR condition than in the long-term EMDR-DBT condition. However, taking into account the whole follow-up period, the clinical efficacy and cost-effectiveness of the long-term EMDR-DBT could still be greater compared to the short-term EMDR treatment, hence the inclusion of a long-term follow up assessment [98, 99]. Last of all, given the clinical nature of the study, blinding of clients and therapists to treatment condition is not possible. Therefore, blinding is partly maintained by ensuring that clients are blinded to research hypotheses and by ensuring that the research assistants who perform the measurements are masked to the client’s treatment condition. The current randomized controlled design therefore constitutes the most rigorous and conservative test of the effects of integrated EMDR-DBT and EMDR-only.

In sum, this is the first study comparing the clinical efficacy and cost-effectiveness of integrated EMDR-DBT and EMDR-only in patients with PTSD and comorbid (sub)clinical BPD, while simultaneously identifying individual predictors and mediators of the treatment response. Results will reveal which treatment works best for this group of patients and will aid in guiding individual treatment choices, thereby personalizing psychiatry.

## Data Availability

The investigator ensures that the client’s anonymity is maintained by assigning a personal identification code to each client, starting with 45 (year of liberation after World War II) followed by number 001, resulting in numbers 45001, 45002 etcetera. Blood and hair samples at T0 are also identified with a personal identification code, starting with a ‘B’ for the blood sample and an ‘H’ for the hair sample. Personal identification codes at T2 match the T0 codes and start with ‘T2’. All documents will be identified by this identification code, not by client’s names or clinical number. The identification codes are matched with client’s names on a Subject Identification Code List, which the investigator will keep in confidence. Personal data will be kept separately from the experimental data acquired. Study outcomes will be reported anonymously. Handling of personal data will comply with the Dutch Personal Data Protection Act. After cessation of the whole study the investigator will maintain study records for 15 years after final publication.

## Abbreviations

BDNF: Brain-derived neurotrophic factor
BPD: Borderline personality disorder
DBT: Dialectical Behaviour Therapy
EMDR: Eye Movement Desensitization and Reprocessing
FKBP5: FK506-binding protein
fMRI: Functional magnetic resonance imaging
GR: Glucocorticoid receptor
PE: Prolonged Exposure
PTSD: Posttraumatic Stress Disorder
